# Differences in Geriatric-Focused Education Preparation and Beliefs About Providing Care to Older Patients in Ghana

**DOI:** 10.1093/geroni/igab046.1351

**Published:** 2021-12-17

**Authors:** Grace Karikari, Samuel Asante

**Affiliations:** 1 University of North Dakota, Grand Forks, North Dakota, United States; 2 Northeastern State University, Broken Arrow, Oklahoma, United States

## Abstract

With the increasing growth rate of older persons and a rise in related illnesses in Ghana, it is vital that health practitioners are equipped with geriatric-oriented knowledge and expertise to provide the needed services to geriatric patients. The purpose of this study was to examine (i) the differences in the level of geriatric-focused educational preparation between graduating medical and nursing students in a Ghanaian public institution, and (ii) the association between educational preparation and students’ beliefs about providing care to geriatric patients. The study hypothesized that students with more positive beliefs will be linked to higher educational preparation. Descriptive and inferential statistics were conducted (n=136 students). Findings show significant differences in the educational preparation of medical and nursing students (t [134] = -3.790, p < .001). Graduating nursing students, comparatively, had higher educational preparation (M=23.14, SDe3.30) than medical students (M=21.14, SD=2.76). However, there was no significant association between students’ educational preparation and their beliefs about geriatric care. The findings underscore the need for extensive or more focused training. Further, the association between beliefs and educational preparation warrants further exploration. The need to examine the social cultural environment within which the research participants reside are discussed.

